# Supplementary material for: The genome sequence of Zethes insularis Rambur, 1833 (Lepidoptera: Erebidae)
Source: Wellcome Open Res. 2026 Jan 9;11:23. [Version 1] doi: 10.12688/wellcomeopenres.25723.1 (PMC12963832; doi:10.12688/wellcomeopenres.25723.1)
Supplement: Peer review history

# Reviewer report: Reviewer response for version 1

**DOI:** 10.21956/wellcomeopenres.28321.r147526

**Published:** 2026-03-05

**Authors:** Vadim Pisarenco

**Affiliations:** 1 Universitat of Barcelona, Barcelona, Spain

The authors present a high-quality, phased, chromosome-level genome assembly of the poorly studied moth species *Zethes insularis*. To my knowledge, this is the first genome sequence available for this genus. The methods are clearly described and detailed enough to allow replication by others. I have some minor comments and suggestions, outlined below:

 1- In the "Assembly statistics" section, the following sentence is a bit confusing ( "Haplotype 1 was curated to chromosome level, while haplotype 2 was assembled to scaffold level.") considering that both haplotypes are at chr-level as you state later ( "[...] assembled using PacBio and Hi-C data and phased. The result is two curated haplotypes.")

 2- Here, " Z chromosome identified by Merian painting. A haplotypic inversion was observed [...]". You might consider providing a more detailed explanation about how the inversions are detected (I understand that by comparing the distribution of orthologs along the chromosomes). You could also highlight those inversions in Figure 4.

 3- (Typo?) BUSCO percentages sum 100% in Table 4, but do not in Figure 6 (100,37) due to % of Missing BUSCOs (1.31% in Figure 6 vs 0.9% in Table 4).

 4- (Suggestion) If you find it appropriate, you could include a map showing the range of distribution of this species.

Are sufficient details of methods and materials provided to allow replication by others?

Yes

Is the rationale for creating the dataset(s) clearly described?

Yes

Are the datasets clearly presented in a useable and accessible format?

Yes

Are the protocols appropriate and is the work technically sound?

Yes

Reviewer Expertise:

Evolutionary and comparative genomics

I confirm that I have read this submission and believe that I have an appropriate level of expertise to confirm that it is of an acceptable scientific standard.

# Reviewer report: Reviewer response for version 1

**DOI:** 10.21956/wellcomeopenres.28321.r146728

**Published:** 2026-02-20

**Authors:** Iker Irisarri, Raul Contreras

**Affiliations:** 1 Museo Nacional de Ciencias Naturales (MNCN-CSIC), Madrid, Spain; 2 Biodiversity and Evolutionary Biology, Museo Nacional de Ciencias Naturales (Ringgold ID: 16625), Madrid, Community of Madrid, Spain

Wahlberg and colleagues present the haplotype-resolved genome assembly of the moth Zethes insularis, which has very good contiguity and completeness statistics and reaches the quality required by EBP. This is part of the Project Psyche and was sequenced by the Wellcome Sanger Institute team.

 Fig 1. The moth is not very visible because it is covered by the tubes. It might be worth checking if other, better pictures are available.

 It might be a coincidence, but might be worth checking whether it is 2.3% in both cases here: “This reduced the scaffold count by 34.2%, increased the scaffold N50 by 2.3%, and reduced the total assembly length by 2.3%”

 Assembly statistics: 1) It is said that haplotype 1 is curated to chromosome-level and haplotype 2 to scaffold level, but both of them recover the 31 expected chromosomes. Why is that? 2)  “The final assembly has a total length of 862.07 Mb in 214 scaffolds, with 93 gaps, and a scaffold N50 of 29.55 Mb (Table 2).” Please, specify that this refers to haplotype 1.

 Fig. 3 Could you please indicate in the caption which haplotype this refers to?

 Please, check this sentence “Table 4 lists the assembly metric benchmarks adapted from Rhie et al. (2021) the Earth BioGenome Project Report on Assembly Standards September 2024.” Should maybe “Rhie et al. (2021)” be written after 2024?

 Page 7. Space missing in “haplotypes.Z”

Are sufficient details of methods and materials provided to allow replication by others?

Yes

Is the rationale for creating the dataset(s) clearly described?

Yes

Are the datasets clearly presented in a useable and accessible format?

Yes

Are the protocols appropriate and is the work technically sound?

Yes

Reviewer Expertise:

Evolutionary genomics

We confirm that we have read this submission and believe that we have an appropriate level of expertise to confirm that it is of an acceptable scientific standard.
